# Infertility-related stress and its relationship with emotional divorce among Iranian infertile people

**DOI:** 10.1186/s12888-023-05159-z

**Published:** 2023-09-12

**Authors:** Fatemeh Shayesteh-Parto, Seyedeh Batool Hasanpoor-Azghady, Soheila Arefi, Leila Amiri-Farahani

**Affiliations:** 1 Department of Midwifery and Reproductive, School of Nursing and Midwifery, Iran University of Medical Sciences, Tehran, Iran; 2 Department of Midwifery and Reproductive, Nursing and Midwifery Care Research Center, School of Nursing and Midwifery, Iran University of Medical Sciences, Tehran, Iran; 3 Monoclonal Antibody Department, Avicenna Research Institute, Tehran, Iran

**Keywords:** Infertility-related stress, Need for parenthood, Relationship concern, Emotional divorce

## Abstract

**Background:**

Infertility affects different aspects of couples’ lives, so it may cause problems in couples’ emotional relationships by increasing marital conflicts. This study aimed to determine Infertility-related stress and its relationship with emotional divorce among Iranian infertile people.

**Methods:**

We conducted a cross-sectional observational study on 200 infertile people. The research environment was one of the well-equipped infertility centers in Tehran, Iran. Continuous sampling was employed. The data collection tools included a general information form, the Fertility Problem Inventory (FPI), and the Emotional Divorce Scale (EDS).

**Results:**

The findings revealed a significant direct relationship between infertility-related stress and all its subscales with emotional divorce in both infertile women and men. In infertile women, the most concern was the need for parenthood, while the lowest concerns were the relationship and sexual concerns. Multiple linear regression analysis indicated that social and relationship concerns predicted 44% of emotional divorce, with social concern being the more influential factor. In infertile men, the need for parenthood was the most significant concern, while relationship and social concerns were less prominent. Multiple linear regression analysis showed that relationship concern predicted 50% of emotional divorce in infertile men. In both infertile men and women, social and relationship concerns explained 45% of the variance in emotional divorce. Among these two variables, relationship concern had a more impact in predicting emotional divorce. Also, there was no statistically significant difference between women and men regarding infertility-related stress and its subscales, except for sexual concern.

**Conclusion:**

The study highlights the importance of the need for parenthood as a main concern among infertile individuals. Increased infertility-related stress and its subscales contribute to higher levels of emotional divorce among this population. Additionally, relationship concern was the lowest concern in infertile people. But it significantly predicts emotional divorce among infertile individuals.

## Introduction

Infertility is a current social concern due to its wide-ranging psychological, physical, social, and financial consequences [1]. Infertility and its treatment process reduce the quality of married life and weaken the emotional bonds of couples [2]. Globally, approximately 15% of couples suffer from infertility [3]. A systematic review and meta-analysis conducted on 58,746 Iranian participants revealed a prevalence of 5.0% for primary infertility and 2.0% for secondary infertility in Iran [4].

Infertility represents a crisis that affects various aspects of life, and when its treatment using various methods fails and persists, the resulting crisis becomes more significant and chronic [2]. The presence of severe and chronic tensions is detrimental to marriages as it indicates the ongoing crisis and the challenges in adapting to it [5]. Newton et al. categorized infertility-related stress into five dimensions: social concern, sexual concern, relationship concern, rejection of a child-free lifestyle, and the need for parenthood. They have defined rejection of a child-free lifestyle as a negative view of a childless lifestyle and future satisfaction, dependent on having children. The need for parenthood refers to a strong identity with the role of parents in which parenting is the basic principle and the main goal in life [6]. The literature discussed the relationship between these five dimensions sparsely with relationships and marital compatibility. Most studies on the psychosocial aspects of infertility highlight the negative impact of infertility on marital conflicts [7–9].

In developing countries, there is a belief that couples without children are infertile. Thus, infertility is a menace to marital stability in these countries [7]. Infertility may cause couples to avoid interacting with people, especially friends who are pregnant or have children. However, they cannot wholly prevent the conflicts that infertility causes in their marital relationships [2]. Infertility-related stress directly or indirectly harms the marital relationship of infertile people and may lead to divorce [10]. Emotional divorce often precedes formal divorce [11]. Guttman’s model explains that, in the first stage of emotional divorce, couples realize the seriousness of their marital problems and feel that their marriage has reached an “unfortunate point”. In the second stage, couples conclude that talking to their spouse is useless, so they should rely more on themselves. In the third stage, they understand they have no relationship with their spouses and do most of their activities alone. Finally, although they are still married, their lives are like those of singles [12]. Research indicates that conflicts and arguments are not direct causes of dysfunctional marriages leading to divorce. Instead, a decline in emotions and feelings diminished positive emotional relationships, and increased sensitivity between couples predict the collapse of married life [13].

Lillard and Wait showed that parenthood is a protective factor versus divorce and separation [14]. Even if a relationship is strengthened by infertility, it cannot eliminate the risk of divorce for infertile couples due to the strong human desire to reproduce [15]. Emotional divorce is more prominent in non-western countries, mainly due to cultural barriers that compel individuals to remain together despite dissatisfaction, particularly in Asian countries like Iran [16]. Several studies have found a significant association between infertility and the number of its treatments and emotional problems [17–19]. However, contrasting results have been reported, such as more sexual and marital satisfaction of infertile couples compared to fertile couples [20], no difference in the quality of life between infertile and fertile couples [21], and no difference in the mean satisfaction score of marriage in two fertile and infertile groups [22]. Other studies also reported that infertility brings some infertile couples closer together and supports each other in the psychological consequences of infertility [2, 23]. According to the different results of these studies, the present study aimed to determine infertility-related stress and its relationship with emotional divorce among Iranian infertile people, which may provide a more comprehensive understanding of the topic.

## Methods

This cross-sectional observational study was conducted on 200 infertile people (100 women and 100 men independent of each other) referring to the subspecialty center for infertility treatment and research of Bahman Hospital in Tehran (capital of Iran), Iran. The center receives approximately 5,000 visitors from various parts of Iran annually.

We estimated the sample size of 194 subjects with a 95% confidence interval and 80% test power considering a Pearson correlation coefficient of 0.2 between Infertility-related stress and emotional divorce. The formula used to calculate the sample size was $n = \frac{{{{({z_{1 - \alpha /2}} + {z_{1 - \beta }})}^2}}}{{{\omega ^2}}} + 3$; $\omega =\frac{1}{2}\text{l}\text{n}\frac{1+\text{r}}{1-\text{r}}$

The infertile individuals referred to the infertility treatment center were almost evenly distributed between genders, so the researchers continuously selected 200 participants in a one-to-one ratio for both sexes. Due to variations in the number of visitors at the infertility center on different days caused by various treatment procedures, we did sampling on all days of the week, one week, even days and another week, odd days. We decided by lottery whether the first week would be odd or even. The sampling period lasted from March 2021 to November 2021. Due to incomplete questionnaires, the response rate for the survey was 90%.

The inclusion criteria for the study were as follows: participants had to possess minimum reading and writing literacy to complete the questionnaires, have confirmed primary or secondary infertility diagnosed by a gynecologist or urologist, have no living child from secondary infertility, have at least one year passed since the diagnosis of infertility, not have endometriosis (for women), not have adopted children, not have any other medical illnesses unrelated to infertility, not have any mental illnesses requiring treatment based on self-report, and not be remarried. The data collection tools included a general information form, the Fertility Problem Inventory (FPI), and the Emotional Divorce Scale (EDS).

### The general information form

It consisted of two parts; the first was demographic information like age, education, occupation, and marriage duration, and the second part of the characteristics of infertility like infertility duration, duration of infertility treatment, current treatment, and the number of previous treatments.

### Fertility problem inventory (FPI)

Newton et al. developed the FPI. It measures the level of infertility-related stress in individuals. The FPI comprises 46 items categorized into five subscales: (1) social concern, referring to the awareness of other’s comments about infertility, as well as feelings of isolation and alienation from family and friends; (2) sexual concern, focuses on diminished sexual enjoyment or self-esteem, along with challenges encountered during scheduled intercourse, which may be typical for couples attempting to conceive; (3) relationship concern, indicates a problem talking about infertility with one’s partner and worries about the effects of infertility on the relationship; (4) need for parenthood, referring to a strong identification with the parental role and perceptions of parenthood as an essential life goal, and (5) rejection of a childfree lifestyle describes a negative attitude towards a life without children and a belief in the future happiness that having a child would bring. Items are rated on a 6-point Likert scale ranging from 1 (strongly disagree) to 6 (strongly agree). Newton et al. found high internal consistency for both the overall measure (α = 0.94) and the subscales (αs ranging from 0.77 to 0.93). Test-retest reliability after a 30-day interval was 0.83 for women and 0.84 for men. A higher score on each scale/subscale indicates higher stress [6]. Samani et al. provided reliability and validity of the Persian version of this questionnaire. They reported high internal consistency for the overall scale (α = 0.86) and the subscales (αs from 0.76 to 0.95). The reliability of the tool for stability with Intra class correlation (ICC) was 0.85 for the whole scale [24]. In the present study, the internal consistency with Cronbach’s alpha coefficient was 0.89 for the overall scale and 0.75 to 0.93 for the subscales. The reliability of the tool with ICC was 0.92 for the whole scale.

### Emotional divorce scale (EDS)

Gutman designed EDS to assess emotional divorce with 24 items. Items were scored with a two-option scale of yes (1) and no (0). After summing up the positive answers, if the number is equal to eight and above, it means that the person lives with emotional divorce [12]. Samari and Nakhaee used this questionnaire in a psychometrics study. Exploratory factor analysis with 466 Iranian men and women showed that all 24 items were in one factor. The factor loading of items was in the range of 0.34 to 0.78. The reliability of the whole scale using Cronbach’s alpha was 0.94 [25]. In the present study, the internal consistency of the scale with Cronbach’s alpha coefficient was 0.92) and the reliability with ICC was 0.95.

Sampling began after approval of the project by the ethics committee of the Iran University of Medical Sciences with the code (IR.IUMS.REC.1399.1012). After explaining the objectives of the study and the principle of confidentiality, the researcher obtained informed written consent from eligible subjects. Then the participants completed the questionnaires by self-administered in one step. We analyzed the data using Pearson correlation and multiple linear regression by SPSS software version 22. The multiple linear regression model was used to determine the simultaneous effect of the variables, each of which was related to the dependent variable using the Enter method. We checked the regression assumptions, such as normality of the residuals, homoscedasticity, multicollinearity, and independence of the residuals, before the performance of the multivariate analysis. The significance level for all tests was p < 0.05.

## Results

Infertile women had a mean [± SD] age of 34.14 [± 5.57] years, a mean [± SD] marriage duration of 7.90 [± 4.71] years, a mean [± SD] infertility duration of 5.43 [± 4.09] years with a range of 1–20 years, and mean duration of infertility treatment of 3.93 [± 3.26] years with a range of 1–15 years.

Infertile men had a mean [± SD] age of 37.43 [± 5.84] years, a mean [± SD] marriage duration of 7.49 [± 4.19] years, a mean [± SD] infertility duration of 4.95 [± 3.84] years with a range of 1–20 years, and mean duration of infertility treatment of 3.72 [± 2.93] years with a range of 1–15 years. More information about the demographic and infertility characteristics of the subjects is presented in Table 1.

**Table 1:** Demographic and infertility characteristics of participants

Characteristics of infertile women (n = 100)	N (%)	Characteristics of infertile men (n = 100)	N (%)
Age (years)		Age (years)	
< 30	27 (27)	< 30	14 (14)
30–40	57 (57)	30–40	60 (60)
> 40	16 (16)	> 40	26 (26)
**Education**		**Education**	
< High school	6 (6)	< High school	2 (20)
High school	25 (25)	High school	20 (20)
Academic	69 (69)	Academic	78 (78)
**Occupation**		**Occupation**	59 (33.3)
Housewife	46 (46)	Employee	47 (47)
Employed	54 (54)	Free	36 (36)
		manual worker	16 (16)
		Unemployed	1 (1)
**Economic status**		**Economic status**	
Favorable	25 (25)	Favorable	30 (30)
Relatively favorable	55 (55)	Relatively favorable	55 (55)
Undesirable	20 (20)	Undesirable	15 (15)
**Marriage duration (years)**		**Marriage duration (years)**	
< 5	41 (41)	< 5	43 (43)
5–10	37 (37)	5–10	36 (36)
> 10	22 (22)	> 10	21 (21)
**Infertility duration (years)**		**Infertility duration (years)**	
< 5	56 (56)	< 5	58 (56)
5–10	35 (35)	5–10	34 (34)
> 10	9 (9)	> 10	8 (8)
**Duration of infertility treatment (years)**		**Duration of infertility treatment (years)**	
< 5	77 (77)	< 5	74 (74)
≥ 5	23 (23)	≥ 5	26 (26)
**Causes of infertility**		**Causes of infertility**	
Female factor	46 (46)	Male factor	46 (46)
Mixed factor	27 (27)	Mixed factor	23 (23)
Unexplained factor	27 (27)	Unexplained factor	31 (31)
**Type of infertility**		Type of infertility	
Primary infertility	52 (52)	Primary infertility	62 (62)
Secondary infertility	48 (48)	Secondary infertility	38 (38)
**Current treatment**		**Current treatment**	
Drug	19 (19)	Drug	22 (22)
^a^IUI	13 (13)	^a^IUI	17 (17)
^b^ICSI or^c^ IVF	68 (68)	^b^ICSI or ^c^IVF	61 (61)

^*^Intrauterine insemination; ^**^Intracytoplasmic sperm injection; ^***^In vitro fertilization

**Table 2:** Descriptive statistics and comparison of the FPI subscales in infertile men and women

Subscales of FPI	Infertile women (n = 100)		Infertile Men (n = 100)		All infertile people (n = 200)		^**^P-value	^***^d
	**Mean (SD)**	^*^Mea**n score based on 100**	**Mean (SD)**	^*^ **Mean score based on 100**	**Mean (SD)**	^*^ **Mean score based on 100**		
**Social concern**	29.77 (9.93)	39.54 (19.87)	29.33 (9.86)	38.66 (19.72)	29.55 (9.87)	39.10 (19.75)	0.754	0.04
**Sexual concern**	21.43 (8.12)	33.57 (20.31)	24.31 (8.48)	40.77 (21.2)	22.87 (8.41)	37.17 (21.02)	0.015	-0.34
**Relationship concern**	26.78 (9.4)	33.56 (18.81)	28.79 (10.65)	37.58 (21.3)	27.78 (10.07)	35.56 (20.14)	0.159	-0.20
**Rejection of a child-free lifestyle**	27.01 (7.9)	47.52 (19.76)	27.15 (6.51)	47.87 (16.28)	27.08 (7.22)	47.7 (18.06)	0.891	-0.01
**Need for parenthood**	37.06 (11.59)	54.12 (23.19)	36.24 (10.95)	52.48 (21.9)	36.65 (11.25)	53.3 (22.51)	0.608	0.07
**Total infertility-related stress**	142.05 (39.97)	41.76 (17.38)	145.82 (38.44)	43.4 (16.71)	143.93 (39.16)	42.58 (17.02)	0.497	0.09

* Since the number of items in the subscales of FPI was different from each other, to compare, we calculated the mean based on 100; **Independent t-test; ***Cohen’s d

Table 2 shows that the subscale of the need for parenthood had the highest mean score based on 100 in infertile women and men, thus in all infertile people.

**Table 3:** Comparison of the FPI subscales according to the type of infertility

Subscales of FPI	Primary infertility (n = 114)	Secondary infertility (n = 86)	^*^P-value	^**^d
	Mean (SD)	Mean (SD)		
**Social concern**	28.92 (9.36)	30.37 (9.79)	0.308	-0.14
**Sexual concern**	22.72 (8.02)	23.05 (8.93)	0.784	-0.03
**Relationship concern**	27.64 (9.97)	27.96 (10.25)	0.827	-0.03
**Rejection of a child-free lifestyle**	27.18 (6.98)	26.94 (7.57)	0.815	0.03
**Need for parenthood**	35.91 (11.19)	37.62 (11.33)	0.287	-0.15
**Total infertility-related stress**	142.40 (39.35)	145.96 (39.04)	0.526	-0.09

*Independent t−test; **Cohen’s d

Table 3 shows that there is no statistical difference in the FPI subscales between the two groups of people with primary and secondary infertility.

The relationship between subscales of FPI and emotional divorce is presented in Table 4.

**Table 4:** Correlation between subscales of FPI and emotional divorce in participants

Subscales of FPI	Infertile women (n = 100)		Infertile Men (n = 100)		All infertile people (n = 200)	
	r	P	r	P	r	P
**Social concern**	0.62	< 0.001	0.56	< 0.001	0.59	< 0.001
**Sexual concern**	0.55	< 0.001	0.52	< 0.001	0.52	< 0.001
**Relationship concern**	0.58	< 0.001	0.69	< 0.001	0.62	< 0.001
**Rejection of a child-free lifestyle**	0.42	< 0.001	0.30	< 0.001	0.37	< 0.001
**Need for parenthood**	0.47	< 0.001	0.44	< 0.001	0.45	< 0.001
**Total stress**	0.62	< 0.001	0.63	< 0.001	0.62	< 0.001

**Table 5:** Results of multiple linear regression analysis to investigate the effect of subscales of FPI emotional divorce in participants

Independent Variable Infertile women (n = 100)	B Coefficient	Standardized Coefficient	Pa	VIF	R2
**Social concern**	0.17	0.36	0.020	4.05	0.44
**Sexual concern**	0.04	0.07	0.556	2.86	
**Relationship concern**	0.15	0.29	0.013	2.26	
**Rejection of child-free lifestyle**	-0.03	-0.05	0.683	2.55	
**Need for parenthood**	0.01	0.03	0.793	3.22	
**Independent Variable** **Infertile Men (n = 100)**	**B Coefficient**	**Standardized** **Coefficient**	**Pa**	**VIF**	**R2**
**Social concern**	0.05	0.12	0.366	3.30	0.50
**Sexual concern**	-0.006	-0.01	0.925	3.05	
**Relationship concern**	0.24	0.59	< 0.001	2.25	
**Rejection of child-free lifestyle**	-0.01	-0.01	0.84	1.72	
**Need for parenthood**	0.02	0.06	0.56	2.63	
**Independent Variable** **All infertile people (n = 200)**	**B Coefficient**	**Standardized** **Coefficient**	**Pa**	**VIF**	**R2**
**Social concern**	0.13	0.27	0.005	3.43	0.45
**Sexual concern**	-0.006	-0.01	0.903	2.79	
**Relationship concern**	0.19	0.41	< 0.001	2.21	
**Rejection of child-free lifestyle**	-0.01	-0.02	0.753	2.01	
**Need for parenthood**	0.03	0.07	0.421	2.81	

We entered all FPI subscales into the multiple linear regression model with the Enter method. For infertile women, the mean emotional divorce score increased by 0.17 units for each unit added to social concern and increased by 0.15 units for each unit added to relationship concern. The social and relationship concern predicted 44% of the changes in emotional divorce. Among these two variables, social concern had a more impact on emotional divorce. In infertile men, the mean emotional divorce score increased by 0.24 units for each unit added to relationship concern, it predicted 44% of the changes in emotional divorce. In All infertile people, the mean emotional divorce score increased by 0.13 units for each unit added to social concern and increased by 0.19 units for each unit added to relationship concern in infertile people. The social and relationship concern predicted 45% of the changes in emotional divorce in infertile people. Among these two variables, relationship concern had a more impact (Table 5).

## Discussion

The present study aimed to determine Infertility-related stress and its relationship with emotional divorce among Iranian infertile people. Findings showed that the highest mean score was the need for parenthood for both infertile men and women. On the other hand, the lowest mean scores were in the relationship concern and sexual concern subscales for women and relationship concern for men. Furthermore, this study revealed a significant direct relationship between infertility-related stress and all its subscales and emotional divorce. Also, relationship concern have more effectiveness in predicting emotional divorce in infertile people.

Comparison with Other Studies: a study on French infertile people showed that the mean infertility-related stress was 145.43 in infertile women and 136.53 in infertile men [26]. In this study, the researchers did not report the mean score of each subscale by gender. The results for women were almost similar to the present study. But the mean score of infertile men was lower than in the present study. Perhaps one of the reasons is the presence of 18% of secondary infertility with one or two children in the mentioned study. Another reason is the experience of infertility in developed and developing societies. In developed societies like France, not having children is voluntary and is considered a suitable option for growth and development. People without children in developed countries are often assumed to be childfree as people who voluntarily choose not to have children [7], while infertility in developing countries (such as Iran) has direct social, psychological and economic consequences [27]. The similarity of results in our infertile women with French infertile women may be due to the high level of education in both groups. In our study, 69% of infertile women had academic education. Scientific evidence suggests that infertile women with higher education may have more social resources, thus enabling them to cope with infertility-related stress and protect themselves [28]. Another study on 410 infertile Iranian individuals (237 women and 168 men) showed a mean infertility-related stress score of 162.23 for both men and women [24]. In the mentioned study, the researchers did not report the mean score of subscales by gender. In this study also, the subscale of the need for parenthood had the highest mean. But the mean score of the infertility-related stress and its subscales were higher compared to the present study, except for the rejection of a child-free lifestyle. Possible reasons for this disparity include differences in sample size, the causes of infertility, and the level of education. The participants with academic education in the current study were 33% more than the mentioned study. Zurlo et al. emphasize education as an essential variable for two reasons. The first reason is related to the greater understanding of infertility and the control of medical treatments. The second reason is due to other happy aspects of life other than motherhood that is paid attention to today [29]. On the other hand, the level of education of the infertile person affects how to deal with infertility. People with higher education use problem-solving coping strategies more than passive strategies due to their ability to search for information sources [2].

A study conducted in Vietnam on infertile women showed a mean infertility-related stress score of 164.20. Similar to the present study, the need for parenthood had the highest mean, while relationship concern and sexual concern had the lowest mean score. However, the mean score of infertility-related stress and its subscales in the Vietnamese study were higher. Possible reasons for these differences include the difference in sample size, mean age and culture. Infertile Vietnamese women may be under more pressure than infertile Iranian women culturally. Truong et al. reported that the sociocultural context in Vietnam exerts severe pressure on infertile women. Common proverbs in Vietnam, titled “More children, more wealth” or “Unblessed trees do not bear fruit, unblessed women do not bear children” show the importance of having children in Vietnamese culture and the much greater stress of Vietnamese women. [30]. Ngai & Loke conducted a study on infertile couples in China. The findings showed that the mean score of infertility-related stress and some of its subscales were slightly higher than the present study [31]. Perhaps one of the reasons for this increase is the impact of unique aspects of Chinese culture on the negative attitude towards infertility. China is the birthplace of Confucianism, which considers a person part of the blood lineage. Being a member of a blood lineage is much more important than the value of a person, and maintaining the lineage is a priority. This cultural legacy remains strong in modern China, putting significant pressure on the family to produce new children and keep the lineage alive, especially under the one-child policy. Although the policy has recently been relaxed to allow two children per family, infertile women are still under a lot of pressure. Traditional Chinese values make infertile people unacceptable, as it represents the disappearance of a generation that has been protected for many generations [32].

The noteworthy point in all the mentioned studies and the present study was the high mean score of the need for parenthood subscale in the studies conducted in both developing and developed countries. Undoubtedly, many factors motivate the need for parenthood. In economic models, children are an economic necessity and generate income as a workforce. In contrast, children in the Western world largely drain the family’s economic resources. They are probably a social investment that confers parental status and creates a normative family unit. They are also a stake in the future and may inherit the property. It is important to note that social factors also play a significant role in shaping the meaning of parenting. Motherhood, for example, has traditionally been viewed as the primary role for women, and infertile women may feel excluded from motherhood. For men, fatherhood can serve as a confirmation of their masculinity and fulfil identity needs. In some societies, particularly in the Western world, social and personal identity needs are strong motivators for parenthood [2, 30, 33, 34]. Scientific evidence shows that although infertile women generally experience more emotional problems than infertile men [35, 36], there is no difference between the two genders regarding parenting needs [36, 37]. The present study also showed that there was no statistically significant difference in the need for parents between infertile men and women. Modern evolutionary psychologists place the need for parenthood at the top of the pyramid for humans [38] and infertility as an obstacle to achieving the goal of parenting life [28, 39]. Scientific evidence also demonstrates that, despite changing family values in the present world, both men and women still highly value the experience of being parents, as it correlates with personal satisfaction, social acceptance, and gender identity [2, 40].

The researchers conducted a comprehensive search and found no existing studies that specifically investigated the correlation between infertility-related stress and emotional divorce. To make comparisons, they relied on studies that explored the relationship between infertility-related stress and other factors such as marital satisfaction, marital compatibility, quality of life, family cohesion, and sexual satisfaction.

Numerous studies conducted in China on infertile women have revealed a detrimental relationship between infertility-related stress and life satisfaction, marital satisfaction [28], marital quality [18], and quality of life [31]. Van Der Merwe and Greeff found a significant negative relationship between infertility-related stress and four aspects of the marital relationship including communication quality, sexual satisfaction, intimacy, and marital adjustment in infertile people [41]. A review study also showed that infertility-related stress is directly related to marital dissatisfaction and poor marital communication [42]. In another study on infertile couples, the results showed that as the infertility-related stress and each of its subscales increased family cohesion and adaptation decreased [43]. It is consistent with the results of the present study.

Nakić Radoš et al. conducted a study in Croatia on infertile couples. They reported a significant negative relationship between infertility-related stress and sexual and relationship concern subscales with sexual satisfaction [44]. Another quantitative study involving infertile men and women in France found that infertility-related stress had predictive effects on emotional distress and marital satisfaction [26]. However, some studies have highlighted that aligned views in infertility stress management can enhance the quality of marital relationships [45], or in infertile men and women who themselves and their spouses have equal levels of perceived social infertility stress, there is a higher level of marital adjustment [46].

### Research limitations

- We collected the data based on self-administered. According to the nature of the research, the cultural factors and values of the society may have influenced the answers to some items.
- The present study was conducted in only one infertility center, which may limit the generalizability of the results, although the number of visitors to this center was from different parts of the country.
- Due to the cross-sectional nature of the present study, it cannot determine the cause-and-effect relationship. Besides, this cross-sectional study was conducted in a one-time frame, different results may be obtained in another time frame.

## Conclusion

The most concern among infertile people was the need for parenthood. As infertility-related stress and all its subscales increase, emotional divorce also rises among infertile individuals. However, relationship concern was the least concern in infertile people. But it had more effective in predicting emotional divorce in infertile people. According to the results, it seems that to reduce emotional divorce in infertile people, more attention should be paid to relationship concerns.

As infertility-related stress and all its subscales increase, the likelihood of emotional divorce also rises among infertile individuals.

## Data Availability

The datasets used and analyzed during the current study are available from the corresponding author upon reasonable request.

## Abbreviations

FPI: Fertility Problem Inventory
EDS: Emotional Divorce Scale
ICC: Intra Class Correlation
IUI: Intrauterine Insemination
ICSI: Intracytoplasmic Sperm Injection
IVF: In Vitro Fertilization
